# Evidence of Higher Oxidative Status in Depression and Anxiety

**DOI:** 10.1155/2014/430216

**Published:** 2014-04-29

**Authors:** G. Grases, M. A. Colom, R. A. Fernandez, A. Costa-Bauzá, F. Grases

**Affiliations:** ^1^Psychology and Neurology Center (CLONUS), 07014 Palma de Mallorca, Spain; ^2^Centro de Enseñanza Superior Alberta Giménez (CESAG), 07013 Palma de Mallorca, Spain; ^3^University Institute of Health Sciences Research (IUNICS-IdIsPa), University of Balearic Islands, 07122 Palma de Mallorca, Spain

## Abstract

We use a simple method for evaluating antioxidative status, by measuring the redox potential of urine, and correlate the findings with measures of anxiety and depression. We include 63 individuals (28 males and 35 females aged between 20 and 65 years). The validated anxiety State-Trait Anxiety Inventory questionnaire and the validated BDI (Beck Depression Inventory) questionnaire were used to evaluate anxiety and depression. Antioxidative status was determined by measuring the redox potential of urine collected in standard conditions. Correlation of the antioxidant capacity of urines evaluated using the ferric ion/specific dye method or through redox potential using the platinum electrode demonstrated the suitability of this last procedure. We found that normal anxiety state values corresponded to low urine redox potentials, whereas higher anxiety states were associated with high urinary redox potential. We also found that individuals with normal BDI values had significantly lower urine redox potentials than individuals with higher BDI values.

## 1. Introduction

Oxidative stress is a consequence of excessive production of free radicals and also due to the failure of the antioxidant defense mechanism that protects the cells removing free radicals [1]. The antioxidant system is integrated by two types of substances: the enzymatic and the nonenzymatic antioxidants [2, 3]. The enzymatic antioxidants are macromolecules (high molecular weight) as superoxide dismutase, glutathione peroxidase, glutathione reductase, glutathione transferase, and catalase. The nonenzymatic antioxidants are little molecules (low molecular weight) as ascorbic acid, vitamin E, uric acid, N-acetyl-cysteine, flavonoids, carotenoids, polyphenols, and phytate. All these little molecules can be filtered by the kidney and can appear in urine. When an excess of free radicals is generated and/or exceeds the antioxidant defense mechanism, this causes important cell damage through alteration of DNA, protein, and lipids and obviously this causes a consumption of antioxidants and oxidative stress appears [1, 4].

Oxidative stress has been associated with the pathogenesis of several diseases [5, 6]. Thus oxidative stress may be a pathogenic factor in many psychiatric disorders [7, 8] due to the high oxygen consumption and lipid-rich constitution of the brain [9, 10] and has recently been implicated in depression and anxiety [11, 12]. Depression and anxiety are related to lowered plasma concentrations of antioxidants, such as vitamin E, tryptophan, tyrosine, albumin, zinc, glutathione, and CoQ10, and lowered antioxidative enzyme activities [11–13]. The deficit of antioxidants can decrease the protection against reactive oxygen species (ROS) and reactive nitrogen species, inducing oxidative and nitrosative stress and damage to fatty acids, proteins, DNA, and mitochondria [11]. The 8-hydroxy-2′-deoxyguanosine in urine is a biomarker for oxidative DNA damage [14, 15]. Thus it has been demonstrated that major depression is accompanied by increased 8-hydroxy-2′-deoxyguanosine levels, indicating oxidative damage to DNA by ROS [16–18]. Increased ROS in depression is also demonstrated by increased levels of malondialdehyde and arachidonic acid [11]. There is also evidence that major depression causes (auto)immune responses [19]. Thus depression is associated with increased levels of plasma IgG antibodies against oxidized LDL and increased IgM-mediated immune response against membrane fatty acids (e.g., phosphatidyl inositol, oleic, and palmitic) and NO modified amino acids [11]. A high anxiety level in mice was associated with significant increase in the generation of ROS by peripheral blood lymphocytes, granulocytes, and monocytes [20]. Other studies demonstrated an increased oxidative stress into the brain of rats subjected to chronic mild stress [21].

These facts introduce new potential targets for therapeutic actions based on antioxidant compounds [12]. Thus, N-acetyl-cysteine has been demonstrated to exhibit significant benefits on depressive symptoms in a randomized placebo-controlled trial. Curcumin also presented antidepressant activity in several animal models of depression, due to its ability to inhibit monoamine oxidases [12]. Treatment with statins, which have potent in vitro and in vivo anti-inflammatory and antioxidative activities, has been associated with significant reduction in the risk of depression in individuals who have had a cardiac event, further supporting the role of oxidative and inflammatory processes in depression [22].

In this paper we measure the urinary redox potential with the aim to evaluate the global antioxidative status and to correlate these findings with measures of anxiety and depression in 63 individuals.

## 2. Material and Methods

Sixty-three volunteers (28 males and 35 females), ranging in age from 20 to 65 years, were selected by the Psychology and Neurology Center (CLONUS). These individuals were recruited by CLONUS among patients and volunteers that accomplished the following inclusion and exclusion criteria.

- Patients with severe mental health disorders (e.g., schizophrenia, major depression, bipolar disorder, and obsessive-compulsive disorder) were excluded. Only patients with a diagnostic of anxiety, depressive disorders, marital conflicts, or behavioral problems were included.
- Participants consuming antioxidant supplements or omega-3 PUFAs were excluded.
- Participants with severe health problems (e.g., cancer, serious cardiopathy) that need chronic pharmacologic treatment were excluded.
- Participants with addiction to alcohol or drugs were excluded.

All participants were not undergoing pharmacological treatment during urine collection and were on an unrestricted diet during urine collection.

All participants were Caucasian and belonging to median-high social status. Each participant provided written informed consent, and the institutional review board of the Balearic Islands Community approved the study (number IB 1912/12 PI).

### 2.1. Anxiety and Depression Questionnaires

The validated anxiety State-Trait Anxiety Inventory (STAI) questionnaire [23] was used to evaluate state (STAIE) anxiety (A/E). A/E < 22,00 were considered normal anxiety state values and A/E ≥ 22,00 were considered high anxiety state values. The validated BDI (Beck Depression Inventory) questionnaire [24] was used to assess depression. BDI < 10,00 were considered normal values and BDI ≥ 10,00 were considered light-moderate depression values.

### 2.2. Measurement of the Antioxidant Capacity of Urine Samples

All subjects were on free diet, and none was receiving pharmacological treatment at the time of urine collection. After an overnight fast, two-hour urine samples were collected early in the morning, after discarding the first micturition. This urine was used to measure redox potential because this measurement better represents the urinary basal redox potential since it is less affected by dietary factors. Each urine sample was immediately cooled to room temperature (25°C), and the redox potential was measured using a Crison potentiometer, with a platinum electrode as the working electrode and a saturated calomel electrode as the reference electrode. For comparative purposes, the antioxidant capacity of the urines was also evaluated using ferric ion and a specific dye (1,10-fenantroline) [25, 26].

### 2.3. Statistical Analyses

All statistical analyses were performed using the Statistical Package for Social Sciences (SPSS, version 11.0 for Windows, Chicago, IL, USA). The Kolmogorov-Smirnov test was performed to evaluate the normality of the variables. Anxiety states were classified in two groups: normal (A/E < 22,00) and high anxiety states (A/E ≥ 22,00). Also two depression groups were considered: no depression (BDI < 10,00) and light-moderate depression (BDI ≥ 10,00). Between groups, differences were assessed using parametric Student's *t*-tests for quantitative variables with normal distribution, non-parametric Mann-Whitney *U* tests for quantitative variables with nonnormal distribution, and Chi-square tests for qualitative variables. A *P* value < 0.05 was considered statistically significant.

## 3. Results

**Figure 1:**
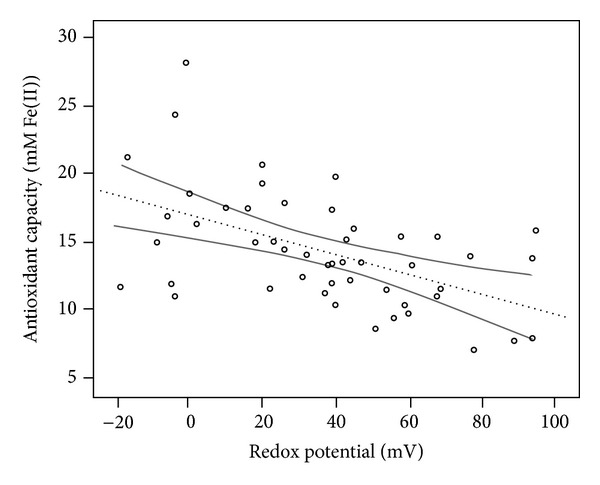
Linear regression between antioxidant capacity (mM Fe(II)) versus redox potential values. The dotted lines are the lines best fit. The solid lines are its 95% of confidence bands. Equation: *y* = −0.073*x* + 16.941, *R* = 0.522, *P* < 0.001.

In Figure 1 the oxidant capacity of urines measured using ferric ion and 1,10-fenantroline and through the direct evaluation of redox potential by means of a platinum electrode is represented. The results demonstrated a good correlation between both measures.

**Figure 2:**
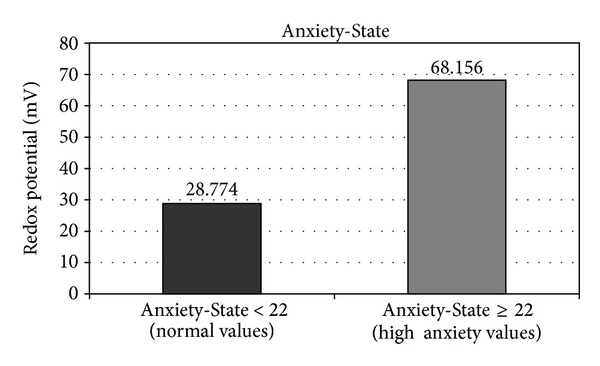
Urinary redox potentials for normal and high anxiety states.

When we measured urine redox potentials in individuals with normal and high anxiety (Figure 2), we found that normal anxiety state values (A/E < 22) corresponded to low mean urine redox potentials (28.8 mV, SD 35,8), whereas higher anxiety states (A/E > 22) were associated with mean urinary redox potential of 68.12 mV (SD 34,8) (*P* < 0.001).

**Figure 3:**
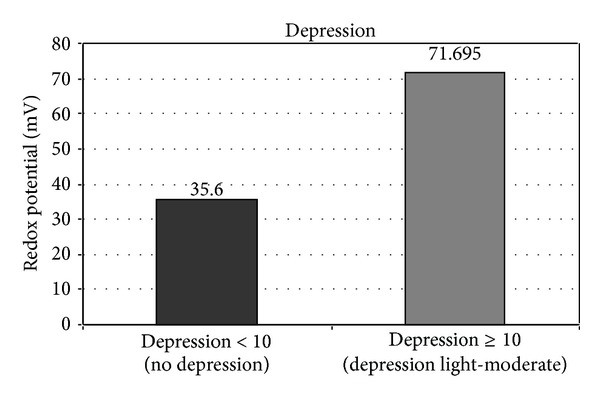
Urinary redox potentials for normal and high depression states.

We also assessed urine redox potentials as a function of depression (Figure 3). We found that individuals with normal BDI values had significantly lower mean urine redox potentials than individuals with higher BDI values (35.6 mV, SD 36,27 versus 71.7 mV, SD 36,9, *P* < 0.001).

As can be seen in Table 1, no statistically significant differences were observed between groups of normal or higher anxiety or between groups of normal or higher BDI values.

**Figure 4:**
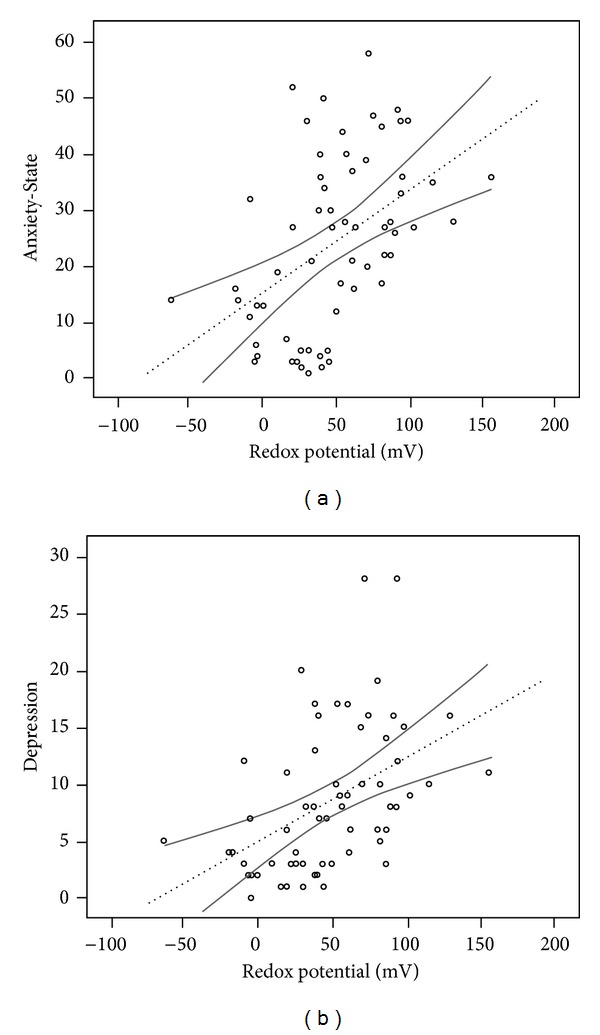
Linear regression between Anxiety-State (a) and Depression (b) versus redox potential values. The dotted lines are the lines best fit. The solid lines are 95% of confidence bands. Equation for Anxiety-State: *Y* = 15.385 + 0.182*X*, *R* = 0.475, *P* < 0.001. Equation for Depression: *Y* = 4.926 + 0.073*X*, *R* = 0.463, *P* < 0,001.

Linear regression between Anxiety-State and Depression versus redox potential was analyzed in each case. The obtained results are shown in Figure 4. As can be seen, in both cases, positive linear relationships were found between both clinical scores and urinary redox potential values (Anxiety-State: *Y* = 15.385 + 0.182*X*, *R* = 0.475, *P* < 0.001. Depression: *Y* = 4.926 + 0.073*X*, *R* = 0.463, *P* < 0,001).

## 4. Discussion

The direct measurement of free radical concentrations is very difficult due to their short half-live and low concentrations. Consequently, measurements of the metabolites of ROS, antioxidant enzyme activities, antioxidant concentrations in blood, and markers of oxidative damage, including DNA damage, lipid peroxidation, and protein carbonylation, are used to quantify levels of oxidative stress [10]. Several procedures are available to determine the global antioxidative capacity of a biological fluid. A commonly used methodology is based on the reaction between the antioxidant present in the sample and ferric ions. The antioxidants present in the sample reduce the ferric ion and the formed ferrous ion complexes a specific dye forming a colored product. Intensity of the color, which is associated to the amount of antioxidant present in the sample, is measured spectrophotometrically [25]. The redox potential of a fluid is a measure of its tendency to act as an oxidizing or reducing media, with a more negative potential indicating a greater reducing capacity or antioxidant activity. In contrast, oxidizing media will have a higher positive redox potential. Due to this, the decrease in the fluid antioxidant status must be reflected by an increase in the fluid redox potential. Correlation of the antioxidant capacity of urines evaluated using the ferric ion/specific dye method [25, 26] or through redox potential using the platinum electrode demonstrated the suitability of this last procedure that was not used before for this purpose. Urine, a product of blood filtration, reflects the composition of blood, including molecules of low molecular weight as vitamins and other antioxidant molecules. Consequently, a deficiency in antioxidants will be reflected in urine composition and the corresponding urinary redox potential, in such a way that individuals with a high oxidative stress will excrete urine with low antioxidant capacity and high redox potential values. The results of this study demonstrate that higher anxiety or depression status of individuals was associated with higher urinary redox potentials. Consequently, our results suggest that individuals with anxiety or depression are deficient in antioxidants, indicative of increased oxidative stress.

## Figures and Tables

**Table 1:** Age distribution (mean and standard deviation) of the different Anxiety-State and Depression groups.

		Age (years)		
	*N*	Mean	SD
Anxiety-State			
<22,00	31	**37,5**	13,7
≥22,00	32	**41,5**	13,1
Depression			
<10,00	40	**38,9**	13,6
≥10,00	23	**40,6**	13,2
